# Ticks and Tick-Borne Pathogens Abound in the Cattle Population of the Rabat-Sale Kenitra Region, Morocco

**DOI:** 10.3390/pathogens10121594

**Published:** 2021-12-09

**Authors:** Latifa Elhachimi, Carolien Rogiers, Stijn Casaert, Siham Fellahi, Thomas Van Leeuwen, Wannes Dermauw, Félix Valcárcel, Ángeles Sonia Olmeda, Sylvie Daminet, Sarah El Hamiani Khatat, Hamid Sahibi, Luc Duchateau

**Affiliations:** 1 Departement de Parasitologie et de Sante Publique, Institut Agronomique et Veterinaire Hassan II, B.P 6202, Rabat 10112, Morocco; fellahisiham2015@gmail.com (S.F.); sahibihamid@gmail.com (H.S.); 2 Biometrics Research Center, Faculty of Veterinary Medicine, Ghent University, Salisburylaan 133, B-9820 Merelbeke, Belgium; carolien.rogiers@ugent.be (C.R.); luc.duchateau@ugent.be (L.D.); 3 Laboratory of Parasitology, Faculty of Veterinary Medicine, Ghent University, Salisburylaan 133, B-9820 Merelbeke, Belgium; Stijn.Casaert@ugent.be; 4 Laboratory of Agrozoology, Department of Plants and Crops, Faculty of Bioscience Engineering, Ghent University, Coupure Links 653, B-9000 Ghent, Belgium; thomas.vanleeuwen@ugent.be (T.V.L.); Wannes.Dermauw@ilvo.vlaanderen.be (W.D.); 5 Plant Sciences Unit, Flanders Research Institute for Agriculture, Fisheries and Food (ILVO), Burg. Van Gansberghelaan 96, B-9820 Merelbeke, Belgium; 6 Grupo de Parasitología Animal, Animalario del Departamento de Reproducción Animal, CN INIA-CSIC, 28040 Madrid, Spain; valcarcel.felix@inia.es; 7 Departamento de Sanidad Animal, Facultad de Veterinaria, UCM, 28040 Madrid, Spain; angeles@ucm.es; 8 Department of Companion Animals, Faculty of Veterinary Medicine, Ghent University, Salisburylaan 133, B-9820 Merelbeke, Belgium; sylvie.daminet@ugent.be; 9 Department of Medicine, Surgery and Reproduction, Hassan II Institute of Agronomy and Veterinary Medicine, B.P 6202, Rabat 10101, Morocco; s.elhamiani@iav.ac.ma

**Keywords:** ticks, tick-borne pathogen, cattle, Morocco, *Hyalomma*, *Rhipicephalus*, *Anaplasma*, *Babesia*, *Ehrlichia*, *Rickettsia*, *Theileria*

## Abstract

Tick-borne pathogens cause the majority of diseases in the cattle population in Morocco. In this study, ticks were collected from cattle in the Rabat-Sale-Kenitra region of Morocco and identified morphologically, while tick-borne pathogens were detected in cattle blood samples via polymerase chain reaction assay and sequencing. A total of 3394 adult ixodid ticks were collected from cattle and identified as eight different tick species representing two genera, *Hyalomma* and *Rhipicephalus*. The collected ticks consisted of *Hyalomma marginatum*, *Hyalomma anatolicum excavatum*, *Rhipicephalus sanguineus sensu lato*, *Rhipicephalus bursa*, *Hyalomma detritum*, *Hyalomma lusitanicum*, *Hyalomma dromedarii*, and *Hyalomma impeltatum*. The overall prevalence of tick-borne pathogens in blood samples was 63.8%, with 29.3% positive for *Babesia*/*Theileria* spp., 51.2% for *Anaplasma*/*Ehrlichia* spp., and none of the samples positive for *Rickettsia* spp. Sequencing results revealed the presence of *Theileria annulata*, *Babesia bovis*, *Anaplasma marginale*, *Theileria buffeli*, *Theileria orientalis*, *Babesia occultans*, *Anaplasma phagocytophilum*, *Anaplasma capra*, *Anaplasma platys*, *Anaplasma bovis*, *Ehrlichia minasensis*, and one isolate of an unknown bovine *Anaplasma* sp. Crossbreeds, females, older age, and high tick infestation were the most important risk factors for the abundance of tick-borne pathogens, which occurred most frequently in Jorf El Melha, Sidi Yahya Zaer, Ait Ichou, and Arbaoua locations.

## 1. Introduction

Ticks and their related diseases are a severe constraint to livestock development in many regions of the world [1,2,3]. Ticks and tick-borne diseases impact cattle production considerably and cause significant losses [4]. The total annual loss in cattle production due to tick-borne diseases in Tanzania was estimated to be 364 million [5], whereas the total annual economic cost related to the abundance of ticks and their transmitted diseases was 308,144 USD in a study conducted on the cattle population of Lake Mburo National Park in South Western Uganda [6].

In Morocco, the tick fauna is diversified [7]. The main reported tick-borne diseases (TBDs) infecting cattle in the country are theileriosis due to *Theileria annulata*, babesiosis caused by *Babesia bigemina*, and *Babesia bovis* and anaplasmosis due to *Anaplasma marginale* infection [8,9,10,11].

Tropical theileriosis (*T. annulata*) is transmitted by ticks of the genus *Hyalomma* [12,13,14]. In endemic areas, tropical theileriosis occurs in summer and spring, following the seasonal dynamics and the geographic distribution of its main vector *Hyalomma detritum* [15,16]. This tick species is well disseminated in North Africa, i.e., Morocco, Algeria, Tunisia, Libya, Egypt, and Sudan [7]. Similarly, bovine babesiosis is a seasonal disease, and its appearance in the field is closely related to the periods of activity of the tick vectors [17]. In Morocco, it has been reported that bovine babesiosis is endemic in all the main dairy cattle areas, and *Boophilus annulatus* is the main vector of this disease [18]. *Anaplasma marginale* is the primary causative agent of bovine anaplasmosis [19]. In the Mediterranean basin, this pathogen is widespread [20,21]. Several tick species are implicated in the transmission of *A. marginale*, e.g., *Boophilus* spp., *Ixodes ricinus*, *Rhipicephalus* spp., *Dermacentor* spp., *Hyalomma* spp., and other vectors, such as insects and the iatrogenic pathway [20].

For decades, no studies have been performed on the presence of ticks and their transmitted pathogens in cattle in Morocco. Furthermore, most of the previous studies on TBPs used serology and microscopy of blood smears. The objective of the present study is to investigate the occurrence of ticks and tick-borne pathogens in the cattle population of the Rabat-Sale-Kenitra region of Morocco using molecular based methods. Moreover, the risk factors of cattle infection by TBPs were investigated, and their zoonotic potential was discussed.

## 2. Results

### 2.1. Prevalence of Ticks

Overall, 81.7% (415/508) of the examined animals were infested with at least one tick. A total of 3394 adult ixodid ticks were collected from cattle. The study showed the presence of two tick genera, namely *Hyalomma* and *Rhipicephalus,* with prevalence of 84.1% (2856/3394) and 15.9% (538/3394), respectively. The collected tick population consisted of 2702 males and 692 females. Females and males represented 19.8% (567/2856) and 80.1% (2289/2856) of *Hyalomma* ticks, respectively, while in *Rhipicephalus* genus, they represented 23.2% (125/538) and 76.8% (413/538), respectively. From these genera, a total of eight species were identified. The most abundant tick species was *Hyalomma marginatum* (38.9%, 1052/2702), followed by *Hyalomma anatolicum excavatum* (33.1%, 894/2702), *Rhipicephalus sanguineus sensu lato* (9.9%, 268/2702), *Rhipicephalus bursa* (5.4%, 145/2702), and *Hyalomma detritum* (4.3%, 116/2702). *Hyalomma lusitanicum* and *Hyalomma dromedarii* were observed with similar low prevalence (3.7%, 101/2702 and 99/2702, respectively), and *Hyalomma impeltatum* had even lower prevalence (1.1%, 29/2702). Data collection according to the location of sampling, species name, and the number of identified male ticks is summarized in Table 1. Tick infestation intensity according to Bush [22] (number of ticks/number of infested animals) was higher in female cattle (5.6) than in males (3.8) and in old and very old animals (6.3 and 6.1, respectively) compared to weaners and adults (2 and 2.9, respectively).

### 2.2. Prevalence of TBPs

The blood samples of all 508 animals were screened for TBPs by conventional PCR with group-specific primer pairs for *Theileria*/*Babesia* spp., *Anaplasma*/*Ehrlichia* spp., and *Rickettsia* spp. The number of PCR-positive samples was 149 (29.3%) for *Theileria*/*Babesia* spp., 260 (51.2%) for *Anaplasma*/*Ehrlichia* spp., and none of the samples was positive (0%) for *Rickettsia* spp. The overall TBP prevalence was 63.8% (324/508) with every individual carrying at least one of the two group pathogens. Eighty-four (25.9%, 84/324) of all infected cattle were found to carry pathogens from the two groups.

All 409 samples positive for genus primers for *Theileria*/*Babesia* and *Anaplasma*/*Ehrlichia* groups were selected for DNA sequencing to identify TBPs species. The number of samples that could be successfully sequenced for at least one of the two groups of pathogens was 304 (74.3%), of which 47 were sequenced for *Theileria*/*Babesia* spp. and 173 for *Anaplasma*/*Ehrlichia* spp., while 84 samples were sequenced for the two groups of pathogens.

In total, 11 different pathogen species or genotypes were identified based on a match in the GenBank database. Ranked according to the most prevalent species, these were: *Babesia occultans*, *Babesia bovis*, *Theileria orientalis*, *Theileria buffeli*, *Theileria annulata*, *Ehrlichia minasensis*, *Anaplasma bovis*, *Anaplasma platys*, *Anaplasma capra*, *Anaplasma phagocytophilum*, *Anaplasma marginale*, and an unknown *Anaplasma* sp. named herewith bovine *Anaplasma* sp.

PCR-positive samples from the *Theileria*/*Babesia* group were detected in all locations, with a high prevalence of 62.5% in JEM (35/56) and a low prevalence of 15.8% in ACH (9/57). Among the 131 positive and sequenced samples, 81 were *T. annulata* (61.8%). Furthermore, sequencing results revealed that 24 animals were infected with *T. buffeli* (18.3%) and 18 with *T. orientalis* (13.7%). *Babesia bovis* occurred in six samples (4.6%) only, all from JEM and KHM. Two cases of *B. occultans* (1.5%) were observed in the JEM and SAY location. Multiple alignments of nucleotide sequences revealed that all the sequences were conserved and shared 99.72% to 100% nucleotide similarity with sequences from GenBank (Table 2).

PCR-positive samples from the *Anaplasma*/*Ehrlichia* group were found mostly in the OLM location in the Middle Atlas (54/63, 85.7%), and the lowest prevalence was observed in the SAY location (11/53, 20.7%). Infections of *A. marginale* were found in all locations, with an overall prevalence equal to 54.1% (139/257). The overall prevalence for *A. phagocytophilum* was 28.8% (74/257), 11.3% (29/257) for *A. capra*, 1.9% (5/257) for *A. bovis,* and 0.3% for bovine *Anaplasma* sp. Infections with *E. minasensis* (2/257, 0.7%) and *A. platys* (7/257, 2.3%) were detected only in OLM. Furthermore, 74.3% (55/74) of *A. phagocytophilum* infections were found in OLM. Alignment of detected pathogen sequences showed that all the previous pathogen sequences were conserved and shared 99.40% to 100% similarity with sequences in GenBank, except for one sequence from a sample collected from OLM, hereafter referred to as bovine *Anaplasma* sp., which shared only 97.38% with an *E. canis* sequence from Iraq (MN227484.1) (Table 3).

Sequencing results showed that co-infections with species from the two studied groups of genera were highest for *T. annulata* and *A. phagocytophilum* (23.8%, 20/84) and common in animals from the Middle Atlas zone (60%, 12/20). Next, co-infections with *T. annulata* and *A. marginale* (19.0%, 16/84) were found mostly in cattle from the Coastal Plains (75.0%, 12/16). Finally, the association of infection with *T. buffeli* and *A. marginale* (17.8%, 15/84) was found at a similar rate in the three agro-climatic zones: five cases in the Middle Atlas and Gharb Plains and six cases in the Coastal Plains (Table 4).

### 2.3. Phylogenetic Analysis and Genetic Distances

**Figure 1:**
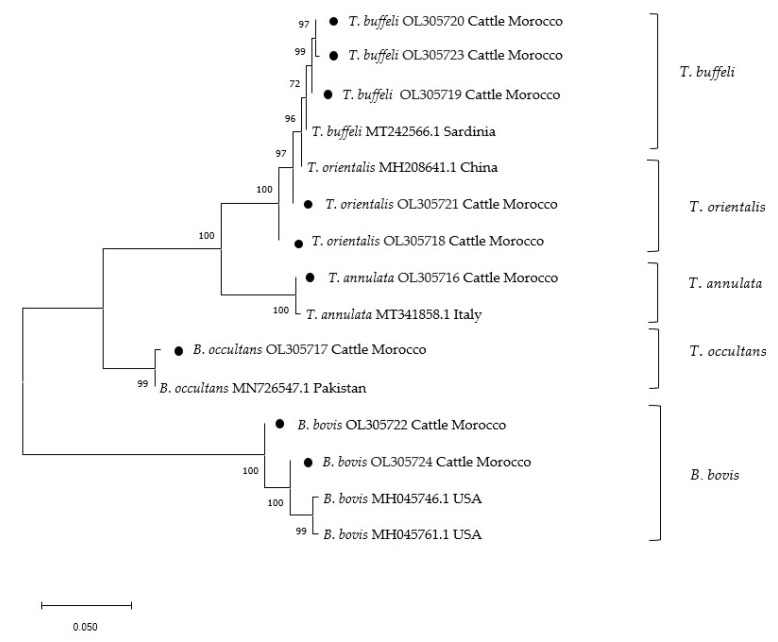
Maximum Likelihood phylogenetic analysis of *Theleria/Babesia* spp. 18S ribosomal RNA gene sequences. Numbers at nodes represent bootstrap support (1000 boutstrap replicates). Sequences obtained during this study are indicated with black dots.

**Figure 2:**
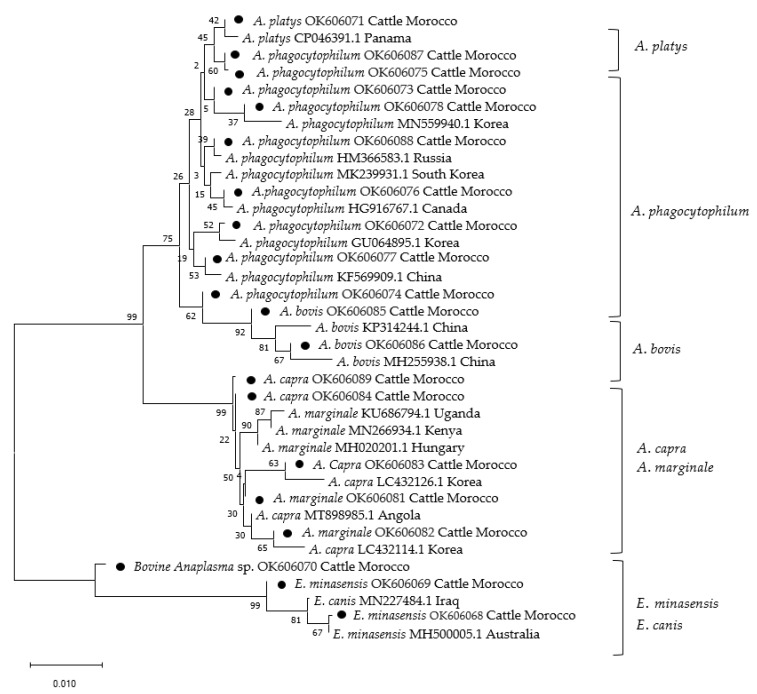
Maximum Likelihood phylogenetic analysis of *Anaplasma/Ehrlichia* spp. 16S ribosomal RNA gene sequences. Numbers at nodes represent bootstrap support (1000 bootstrap replicates). Sequences that were obtained during this study are indicated with black dots.

Maximum likelihood phylogenetic analysis for the genera *Theileria*/*Babesia* and *Anaplasma*/*Ehrlichia* shows the evolutionary relationships of the newly acquired sequences in comparison to published GenBank entries (Figure 1 and Figure 2).

Sequence alignment and phylogenetic analysis were performed by constructing the phylogenetic tree based on 18S ribosomal RNA gene for *T. annulata*, *T. buffeli*, *T. orientalis,* as well as sequences of *B. bovis* and *B. occultans* generated from this study and matched species in the GenBank (Figure 1). Moroccan field isolates *T. buffeli* (OL305720, OL305723, OL305719) and *T. orientalis* (OL305721, OL305718) and matched species in the GenBank, *T. buffeli* from Sardinia (MT242566.1), and *T. orientalis* from China (MH208641.1) shared 100% identity and clustered in the same clade. *Theileria annulata* sequences from this study (OL305716) are 100% identical and appeared in the same clade with *T. annulata* from Italy MT341858.1. Meanwhile, the two sequences of *B. occultans* generated in this study (OL305717) are identical, and sequence analysis showed 100% identity to a reported sequence MN726547.1 from bovine ticks in Pakistan.

Sequences analysis and maximum likelihood phylogenetic analysis for the genera *Anaplasma*/*Ehrlichia* based on the gene sequence 16S rRNA showed the evolutionary relationships of the newly acquired sequences in comparison to published GenBank entries (Figure 2). Generated *A. phagocytophilum* in this study (OK606078, OK606088, OK606076, OK606072, OK606077, OK606073) shared 99.7% to 100% identity with sequences from Korea (GU064895.1, MN559940.1, MK239931.1), Russia (HM366583.1), Canada (HG916767.1), and China (KF569909.1). The new sequences of *A. platys* from Morocco are identical (OK606071) and shared 100% identity with the *A. platys* sequence (CP046391.1) from Panama. The two Moroccan *A. bovis* sequences from this study, OK606085 and OK606086, shared 99.70% and 100% identity with two sequences from China KP314244.1 and MH255938.1, respectively. The acquired *A. bovis* and matched *A. bovis* in the GenBank clustered within the same clade. Phylogenetic analysis showed that all newly generated and matching sequences of *A. phagocytophilum*, *A. platys,* and *A. bovis* clustered within the same monophyletic group. Twenty-one sequences of *A. capra* from this study were identical (OK606083) and shared 100% identity with *A. capra* from Korea (LC432126.1). Other sequences of *A. capra* from Moroccan field samples (OK606084 and OK606089) shared 99.71% with sequences from Korea (LC432114.1) and Angola (MT898985.1). Sequences of *A. marginale* from Moroccan cattle OK606081 and OK606082 shared 100% identity with sequences from Hungary (MH020201.1) and Kenya (MN266934.1), respectively. Acquired and matching sequences of *A. capra* and *A. marginale* formed a monophyletic group. Generated sequences of *E. minasensis* (OK606069 and OK606068) from this study matched very well and clustered in the same clade with corresponding published sequences of *E. minasensis* (MH500005.1) and *E. canis* (MN227484.1) in GenBank, except the sample closely related to *E. canis*, named here as bovine *Anaplasma* sp. (OK606070), which formed a separate branch.

### 2.4. Risk Factor Analysis

The risk factor analysis was only performed for two pathogens, *A. marginale* and *T. annulata*, as the other pathogens had too low prevalence to be analyzed properly.

For *A. marginale*, all studied risk factors except the health status had a significant effect (Table 5). Crossbreeds (compared to local breeds), female (compared to male) animals, modern (compared to traditional) farms, and beef (compared to dairy) cattle were at higher risk for *A. marginale* infections. With age and number of ticks, the risk for *A. marginale* infection declined. Locations also differed significantly from each other, with JEM and SAY having low prevalence and ACH and ARB having the highest prevalence.

For *T. annulata*, all studied risk factors had a significant effect (Table 5). Crossbreeds (compared to local breeds), female (compared to male) animals, traditional (compared to modern) farms, dairy (compared to beef) cattle, and sick (compared to healthy) animals were at higher risk for *T. annulata* infections. With age and number of ticks, the risk for *T. annulata* infection increased. Locations also differed significantly from each other, with OLM and ACH having low prevalence and JEM and SYZ having the highest prevalence.

## 3. Discussion

In the present study, morphological identification of the collected tick specimens revealed the abundance of hard ticks from two genera, *Hyalomma* and *Rhipicephalus,* with eight species, including *H. marginatum*, *H. a. excavatum*, *R. sanguineus s.l.*, *R. bursa*, *H. detritum*, *H. lusitanicum*, *H. dromedarii,* and *H. impeltatum*. Consequently, the Rabat-Sale-Kenitra region is one of the main tick reservoirs in the country. This study showed that *H. marginatum* or the Mediterranean *Hyalomma* is the most prevalent tick species existing in all sampled locations. This tick species is the main vector of Crimean-Congo hemorrhagic fever virus (CCHFV) and is responsible for the transmission of piroplasmosis to cattle [7]. Further studies are required to investigate the competent vector role of this tick species to the detected pathogens in this region.

Based on PCR and sequencing results, we found a high TBP prevalence, including a high level of co-infection with other TBP species from the two groups *Theileria*/*Babesia* and *Anaplasma*/*Ehrlichia*, while none of the cattle samples tested positive for *Rickettsia* spp. Many of the identified TBPs in this study are of major economic importance in North Africa [23,24], while some are causing zoonotic infections in humans. The most common combination found was *T. annulata* and *A. phagocytophilum*, detected in 20 animals. The investigated TBPs differed significantly depending on the cattle breed, age, sex, and geographical location. Cross-breeds, older age, females, and animals from ACH, ARB, JEM, and SYZ locations were the highest risk factor, respectively. The use of molecular techniques allows the detection of pathogens in carrier or sub-clinically infected animals compared to the previous studies in Morocco [25,26].

### 3.1. Theileria/Babesia Group

*Theileria* spp. are hemoprotozoan parasites infecting domestic and wild ruminants. In this group, *T. annulata* is the only species isolated previously in Morocco [15,27]. *Theileria annulata* is a highly pathogenic and host-cell transforming parasite causing tropical theileriosis in cattle with varying degrees of severity [28]. Tropical theileriosis caused by *T. annulata* is the economically most important TBD in North Africa, causing high morbidity and mortality in cattle [23]. In this study, the high overall prevalence of *T. annulata* (26.6%) as compared to other pathogens in the *Theileria/Babesia* group is in line with previous studies in Morocco [9,11]. The higher prevalence may be explained by the ability of cattle to carry this pathogen for a long time after infection without showing signs of illness, which explains the endemic stability. When animals are under physiological stress, this pathogen develops and causes the disease. *Theileria annulata* infection was found in all the study site with high prevalence, even in previously non-endemic locations, like Sidi Yahya Zaer (unpublished data) with high prevalence. This finding may be due to the spreading of the tick vector (*H. detritum*) in previously non-endemic regions because of climate change and an uncontrolled movement of animals in the country. This study is the first report of *T. orientalis* and *T. buffeli* in Morocco. The two pathogens are non-transforming host-cell parasites. These two species are considered as one species by some authors [29,30] and as separate species within a group by others [31]. *Theileria orientalis* and *T. buffeli* were considered benign; however, it has been demonstrated that their virulence is related to their genetic diversity and host specificity [32]. Generally, *Theileria* species cause clinical disease in cattle [33]. Here, *T. buffeli* and *T. orientalis* were detected in asymptomatic cattle with lower prevalence (6.2% and 4.6%, respectively) than *T. annulata*. Lower prevalence was reported in other African countries, e.g., Ethiopia with 1.3% and 0.9% for *T. buffeli* and *T. orientalis*, respectively [34], and Egypt with 0.68% for *T. orientalis* [35]. *Amblyomma* sp. and *Rhipicephalus* sp. are considered as major tick vectors of *T. orientalis* and *T. buffeli* in Africa [36,37]. However, further studies are needed to investigate the role of different tick species in Morocco to transmit those hemoprotozoan parasites to cattle.

Infections with *Babesia* spp. occur in large domestic animal species, including sheep, goats, horses, and dogs, as well humans. Major economic losses are observed in cattle [38]. In this study, the prevalence of *B. bovis* (1.5%) was very low compared to 15.9% in the study conducted earlier in Morocco by Sahibi et al. [39] using serology and blood smears. In the same study, the prevalence of *B. bigemina* was 5.4%, while in our work, none of the tested samples was positive for this pathogen. Similarly, *B. bovis* was detected in cattle from eastern Algeria, while *B. bigemina* was absent in a molecular survey of tick-transmitted protozoa and bacteria [40]. However, in Kenya, both *B. bovis* and *B. bigemina* were reported in cattle using nested PCR and sequencing [41].

This study is the first to report the presence of *B. occultans* from two naturally infected cattle in Morocco. Compared to *B. bovis* and *B. bigemina*, *B. occultans* is characterized by its low pathogenicity in cattle [42]. Recently, a study conducted in Iran has reported that two cases of cows infected with *B. occultans* manifested clinical signs [43]. The absence of isolation and identification of *B. occultans* in previous studies on bovine babesiosis in Morocco may be due to the use of blood smears and serology for TBPs. This study showed that *Hyalomma* ticks are widely distributed, and it was reported that ticks of this genus play an important role in the transmission of *B. occultans* [44,45,46]. Further investigations are warranted to assess the tick vector role associated with these tick-borne protozoa.

### 3.2. Anaplasma/Ehrlichia Species

*Anaplasma* spp. are important tick-borne pathogens of ruminants and humans [47]. Infections with these bacteria may vary from asymptomatic to fatal diseases [48,49]. Our findings confirmed that bovine anaplasmosis is endemic in Morocco, and *A. marginale* is a predominant TBP in cattle, with an overall prevalence of 45.7%. Previous studies in Morocco using microscopic examination of blood smears and serology showed a lower prevalence of *A. marginale* [8]. Moreover, the study conducted in North Central Morocco showed an overall prevalence of 16.5% by competitive enzyme-linked immunosorbent assay (cELISA) and 21.9% by nested polymerase chain reaction (nPCR) assay in cattle [10]. These results are generally lower compared to our finding, and prevalence rates seem to be considerably lower using serological techniques than molecular-based ones. ELISA is based on the detection of antibodies and hence the animal health status and the ability of the immune system to produce detectable antibodies against pathogenic antigens during infection, which highly influences the sensitivity of this technique [26]. Furthermore, our observed prevalences are higher than those observed in neighboring countries, including Algeria (13% by microscopic examination of blood smears) [50] and Tunisia (24.7% by PCR using primer pairs targeting msp4) [51]. The reported overall prevalence of *A. marginale* in cattle was 7.4%, 30.7%, and 0.6% in Tanzania, Cameroon, and Kenya, respectively [52,53,54].

Ruminants are a competent reservoir of *A. phagocytophilum*. This agent of anaplasmosis was described for the first time in ruminants in 1932 [55] and later as an agent of human granulocytic anaplasmosis [56,57]. Tick-borne fever disease due to *A. phagocytophilum* in ruminants and other animal species has been widely reported in Europe [58], while its description in North Africa is recent. Its circulation is currently confirmed in Tunisia, Algeria, and Egypt [21,59]. However, in Morocco, the study conducted by Ait Lbacha et al. [60] did not detect *A. phagocytophilum* in ruminants. The studies conducted by Elhamiani Khatat et al. [61,62,63] showed a high seropositivity rate of *A. phagocytophilum* in humans in Morocco; however, they failed to detect the DNA of the bacterium in dogs. Using conventional PCR and sequencing results, this study confirmed the circulation of *A. phagocytophilum* in Morocco with considerable prevalence in cattle (19.1%). Although *Ixodes* ticks are considered as the main vector of *A. phagocytophilum*, the DNA of this bacterium has been detected in other hard tick species, including *Rhipicephalus* spp. and *Hyalomma* spp. [64,65]. Furthermore, the absence of *Ixodes* ticks in our study area and the abundance of *Hyalomma* and *Rhipicephalus* tick species suggest that studies to investigate the vector role competence of these tick species to transmit *A. phagocytophilum* are needed.

In the group *Anaplasma* spp., it is the first evidence of the presence of *A. capra*, *A. bovis,* and *A. platys* in cattle from Morocco albeit with a very low prevalence. The species *A. capra* was first isolated from goats and humans in China [66,67]. Later, this tick-borne pathogen has been shown to infect sheep, cattle, dogs, and wild animals [68,69,70,71]. Recently, the zoonotic character of *A. capra* was confirmed in vitro by Peng et al. [72]. Thus, *A. capra* and *A. phagocytophilum* are presenting a potential public health threat in many regions of the world as well in Morocco. *Anaplasma bovis* infection was confirmed in diverse animal species, including ruminants, dogs, and cats. In general, *A. bovis* is less pathogenic than other *Anaplasma* species and usually causes subclinical infection in cattle [21]. In North Africa, an identical variant of *A. bovis* was isolated from animals in Tunisia (cattle, sheep, and goats) and Algeria (cattle) [21].

*Anaplasma platys* was first described in dogs from the USA by Harvey et al. [73]. It has been reported to cause infectious canine cyclic-thrombocytopenia as well as infections in cats, cattle, goats, camels, and humans [74,75,76,77,78]. Recently, it has been reported that *Rhipicephalus sanguineus s.l.* is the biological vector of this pathogen [79]. Elhamiani Khatat et al. [64] reported the presence of *A. platys* in dogs from Morocco with 7.5% of prevalence. The high prevalence of collected *R. sanguineus s.l.* from cattle in this study presents a high-risk factor of bovine infection with *A. platys* in the Rabat-Sale-Kenitra region.

The highest rate of infection with *Anaplasma* spp. was detected in the Middle Atlas zone. This finding may be attributed to the extensive forests in this area sheltering wild animals, which are the main reservoirs for tick-borne pathogens [80,81,82].

For a long time, bovine ehrlichiosis was mostly associated with infection by *E. ruminantium*; however, an ehrlichial species, namely *E. minasensis,* which is closely related to *E. canis*, was reported to be infecting dairy cattle and deer in many regions of the world [83,84,85]. A previous study reported the genomic characterization of the bacterium *E. minasensis* isolated from a naturally infected calf with clinical signs of ehrlichiosis [86]. In the study conducted by Cicculli et al. [87], *E. minasensis* was detected in *H. marginatum* ticks collected from cattle in Corsica, France. Our study showed that the Rabat-Sale-Kenitra is an endemic region of *H. marginatum* tick species, which may explain the presence of this bacterium in collected samples. Further studies are needed to investigate the presence of this microbial agent in animals and ticks as well its clinical significance in cattle from Morocco.

For identification at the genus level, the similarity of the 16S rRNA gene sequence should be at least 95% and, for identification at the species level, at least 99% [88]. In this investigation, one field isolate 16S rRNA sequence had only 97.38% similarity with the *E. canis* sequence from Iraq deposited in the GenBank under the accession number MN227484.1. Thus, our results indicate that there is one species within the genus *Anaplasma* that, till now, had not been sequenced and circulates among ruminants in the Rabat-Sale-Kenitra region, Morocco. Because of the limitation of the 16S rRNA gene analysis in the determination of novel taxa [89], further genetic analysis is needed to characterize the unknown species of bovine *Anaplasma* strain observed in this study.

In the genus *Anaplasma*, tick-borne pathogens with zoonotic character are emerging and spreading in many regions of the world. Therefore, a further molecular survey of *Anaplasma* spp. is needed in the country.

In this study, ticks were not analyzed for TBPs, and the information on infected tick vs. the infected cattle is missing, which might lead to an underestimation of the prevalence of TBPs and the risk that ticks represent to human and bovine health.

In Morocco, most of the farms are traditional with a mixture of animal species, e.g., goats, sheep, cattle, and camels. Usually, dogs are present as the companions of the flock. These conditions are a high-risk factor of TBPs infection for animals as well humans.

Finally, this study suggests that theileriosis, babesiosis, as well as anaplasmosis and ehrlichiosis should be considered as important veterinary concerns in the Rabat-Sale-Kenitra region of Morocco, and further research on the dispersion of tick species and molecular assays for detection of infection in ticks and animals to microbial agents is required.

## 4. Materials and Methods

### 4.1. Study Sites and Locations

The study was conducted in the Rabat-Sale-Kenitra region, located in the northwest of Morocco. This region includes the capital of the country, three prefectures—Rabat, Sale and Skhirat-Temara—and four provinces—Kenitra, Khemisset, Sidi Kacem, and Sidi-Slimane.

**Figure 3:**
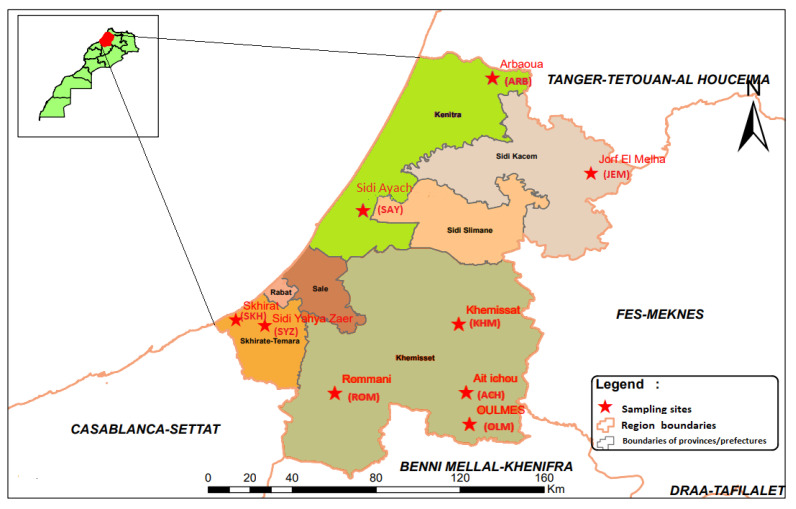
Map of Sampling locations in the Rabat-Sale-Kenitra region of Morocco.

The climate of the region is a semi-arid Mediterranean type with maritime or continental oceanic influence. This region is classified (Csa) according to Köppen-Geiger. Agriculture constitutes the main economic sector of the region (56% of the regional useful agricultural area (UAA) and 12% of the country’s UAA). In addition to agriculture, livestock has an important role, especially intensive cattle farming, with almost 520,000 heads of cattle. This region is including three different agro-climatic zones. The Gharb plains are characterized by a well-developed irrigated system with diversified agriculture and a high concentration of large dairy cattle farms. The coastal plains, where agriculture is based on rains and characterized by increasingly intensive cattle production (milk and beef), has a mixture between subsistence and large farms. The Middle Atlas, a part of Atlas mountainous region, is characterized by traditional farming of cattle and summer transhumance of sheep and goat flocks. Samples were collected from 9 locations well distributed in the different agro-climatic zones in the Rabat-Sale-Kenitra region (Figure 3).

### 4.2. Sample Collection

A total of 508 animals were examined randomly for tick infestation and blood collection in 117 farms belonging to the nine locations (Table 6). A maximum of five animals per farm were selected for sampling. All animals included in the study were sampled with the oral consent of their owners. The sampling was carried out in July and August 2019, the middle of the summer season in Morocco, with typically high tick infestation and TBPs-infection levels.

Information on age, sex, health status, breed, and farm information were obtained through farmer’s records and animal clinical examination. Four hundred and twenty cattle were females, and eighty-eight were males. Different cattle breeds were sampled in different locations, including indigenous Moroccan cattle, referred to as local breed, and their cross-breeds with Holstein, Charolais, and Belgian Blue, referred to as cross-breed. Farms with less than 20 cattle were considered traditional, and if there were more than 20 cattle, they were classified as modern. Cattle were divided into four age groups: weaners (1–2.5 years old), adults (2.5–4.5 years old), old (4.5–8 years old), and very old (>8 years old).

Collected ticks were removed from restrained animals with particular care using forceps on the following predilection sites: ears, neck, udder, perineum, and dewlap. The specimens were put in tubes with 70% ethanol. Immediately upon arrival at the laboratory, ticks were identified under a stereomicroscope according to identifications keys [90,91]. Female ticks were identified at the genus level, while males were identified at the species level.

Approximately 4 mL of whole blood was collected from each selected animal by jugular venipuncture using a sterile vacutainer tube containing ethylenediaminetetraacetic acid (EDTA). Collected samples were transported to the lab in cool boxes and kept at −20 °C until DNA extraction. Ethical approval for this study was obtained from the Ethical Committee for Biomedical Research of the Mohammed V University of Rabat (n°627; July 2019).

### 4.3. DNA Extraction

For each sample, genomic DNA was extracted from the 100 µL EDTA whole-blood sample using the DNeasy Blood Kit (Qiagen, Germany) according to the manufacturer’s instructions and stored at −20 °C until PCR analysis.

### 4.4. PCR Amplification

Conventional PCR with generic primers was used to identify groups of TBPs. To analyze blood samples for the presence of *Theileria*/*Babesia* spp., the genus-specific primers targeting the amplification of the V4 hypervariable region of the 18S ribosomal RNA gene were used (Table 7). Positive controls of *T. annulata* and *B. bigemina* were kindly supplied by colleagues from the Institute of Tropical Medicine Antwerp, Belgium, while double-distilled water was used as a negative control. Thereafter, all samples were screened by PCR for the presence of *Anaplasma*/*Ehrlichia* and *Rickettsia* pathogens using the genus-specific primers for *Anaplasma*/*Ehrlichia* and *Rickettsia* spp., targeting the 16S ribosomal RNA gene (Table 7). Genomic DNA of *A. phagocytophilum* and *E. canis* was obtained from the laboratory of bacteriology, Faculty of Veterinary Medicine, Ghent University Belgium and *Rickettsia massiliae* from the laboratory of parasitology, Faculty of Veterinary Medicine, Ghent University Belgium and was used as positive control, while double-distilled water was served as a negative control.

For PCR reaction, a PCR mix was made in a total volume of 10 µL per sample, containing 2 µL of template, 0.1 µL of TEMPase Hot Start DNA Polymerase (5 U/µL; VWR, Leuven, Belgium), 0.2 µL of dNTP Mix (10 mM each; Bioline, London, UK), 1 µL of the adequate primers (forward and reverse, 5 µM each), 1 µL of 10× Key Buffer (VWR), and 5.7 µL of ultrapure water.

The PCR program consisted of an initial step of 14 min 30 s at 95 °C followed by 40 amplification cycles (denaturation for 30 s at 95 °C, annealing for 30 s at the specific annealing temperature for each primer, and extension for 1 min 30 s at 72 °C) and a final elongation step of 5 min at 72 °C. The corresponding gene loci, selected primer pairs, and annealing temperatures are presented in Table 7. PCR amplicons were subjected to electrophoresis on an agarose gel stained with ethidium bromide and visualized under a UV transilluminator. All positive samples were sequenced to identify the pathogen species.

### 4.5. Sequencing of the PCR Positive Samples

Sequencing reactions were performed using the BigDye Terminator v3.1 Cycle Sequencing Kit (Applied Biosystems, Foster City, CA, USA) and run at Eurofins Genomics (Ebersberg, Germany). The sequence alignments were screened and corrected using BioEdit software version 5.0.9 [92]. The nucleotide sequences were analyzed and compared with available sequences in GenBank using the NCBI’s analysis tool. The open-source BLAST program of the National Center for Biotechnology Information, Bethesda MD (http://blast.ncbi.nlm.nih.gov/Blast.cgi, accessed on 18 February 2021) was used to search for similarity to other sequences in the public database.

### 4.6. Phylogenetic Analysis and Genetic Distances

Phylogenetic analysis and tree construction for the genera *Theileria*/*Babesia* and *Anaplasma*/*Ehrlichia* were generated using open-source molecular evolutionary genetics analysis (MEGA) software Version 10 program [93]. A maximum likelihood phylogenetic tree was reconstructed. The Akaike information criterion was used to select the substitution model, Tamura 3-parameter model with gamma-distributed rates among sites, for maximum likelihood phylogeny. The robustness of the tree was established by bootstrap analysis with 1000 replicates. Bootstrap values above 50 were labeled on major tree branches to assign confidence levels to branches.

### 4.7. Nucleotide Sequences Accession Numbers

Representative gene sequences obtained in this study were submitted to the GenBank database of the National Center for Biotechnology Information using Bankit. The GenBank accession numbers were assigned to the sequenced genes, as shown in Table 8.

### 4.8. Statistical Analysis

The analysis was based on the logistic regression model. First, the location was introduced in the model. Next, each of the covariates was added to this model in a univariate way. Testing was based on the chi-square test, and results were reported in terms of percentage infection. Correlation between the presence of the most prevailing parasites was quantified by Kendall’s tau.

## 5. Conclusions

This study provides insights regarding the tick species and tick-borne pathogens found in cattle from the Rabat-Sale-Kenitra region of Morocco and reinforces the utility of tick-borne pathogens identification by molecular methods. This study revealed high prevalence of *H. marginatum* ticks and diversity of tick-borne pathogens in cattle in the study area with the detection of *Theileria* spp. (*T. annulata*, *T. orientalis*, *T. buffeli*), *Babesia* spp. (*B. bovis*, *B. occultans*), *Anaplasma* spp. (*A. bovis*, *A. capra*, *A phagocytophilum*, *A. platys*), and *Ehrlichia* spp. (*E. minasensis*).

Findings of this study are an important trigger to direct the attention of veterinary and public health stakeholders in Morocco toward ticks and their emerging pathogens.

## Figures and Tables

**Table 1:** Tick species abundance in cattle according to location in the Rabat-Sale-Kenitra region, Morocco.

Zone	Location	*H. marginatum*	*H. a. excavatum*	*H. detritum*	*H. lusitanicum*	*H. dromedarii*	*H. impeltatum*	*R. sanguineus s.l.*	*R. bursa*	Total Ticks (%)
Middle Atlas mountains	ACH	72	173	0	12	6	0	25	7	295 (10.9)
	KHM	78	132	1	7	6	0	30	12	266 (9.8)
	OLM	76	143	3	9	3	0	7	2	243 (9)
The Gharb Plains	ARB	114	81	7	13	0	2	22	14	253 (9.4)
	JEM	188	80	27	6	12	10	62	38	423 (15.6)
	SAY	157	59	28	14	11	14	36	24	343 (12.7)
The Coastal Plains	ROM	60	80	10	20	1	1	28	18	218 (8.0)
	SKH	110	105	20	6	28	0	21	12	302 (11.2)
	SYZ	197	41	20	14	32	0	37	18	359 (13.3)
Total ticks (%)		1052 (38.9)	894 (33.1)	116 (4.3)	101 (3.7)	99 (3.7)	27 (1.1)	268 (9.9)	145 (5.4)	2702 (100)

Abbreviations: *H.*, *Hyalomma*; *a.*, *anatolicum*; *R.*, *Rhipicephalus*.

**Table 2:** *Theileria*/*Babesia* species in cattle blood from the Rabat-Sale-Kenitra region in Morocco, determined by DNA sequencing and matched species in GenBank database.

Strain of Reference	PI (%)	Country	Accession Number	Number of Positives (*n* = 131)
*B. occultans*	100.00	Pakistan	MN726547.1	2
*B. bovis*	99.74	USA	MH045746.1	5
*B. bovis*	99.72	USA	MH045761.1	1
*T. orientalis*	100.00	China	MH208641.1	18
*T. buffeli*	100.00	Sardinia	MT242566.1	24
*T. annulata*	100.00	Italy	MT341858.1	81

Abbreviations: PI, percentage identity (similarity in %) between the field isolate from this study and matched species in the GenBank.

**Table 3:** *Anaplasma*/*Ehrlichia* species in cattle blood from the Rabat-Sale-Kenitra region in Morocco, determined by DNA sequencing and matched species in GenBank database.

Strain of Reference	PI (%)	Country	Accession Number	Number of Positives (257)
*E. minasensis*	99.42	Australia	MH500005.1	2
*A. bovis*	100.00	China	MH255938.1	4
*A. bovis*	99.71	China	KP314244.1	1
*A. platys*	100.00	Panama	CP046391.1	7
*A. capra*	100.00	Korea	LC432126.1	21
*A. capra*	99.71	Korea	LC432114.1	5
*A. capra*	99.71	Angola	MT898985.1	3
*A. marginale*	100.00	Hungary	MH020201.1	131
*A. marginale*	100.00	Kenya	MN266934.1	8
*A. phagocytophilum*	99.70	Korea	GU064895.1	30
*A. phagocytophilum*	100.00	Russia	HM366583.1	23
*A. phagocytophilum*	100.00	Korea	MK239931.1	13
*A. phagocytophilum*	100.00	Canada	HG916767.1	3
*A. phagocytophilum*	99.71	Korea	MN559940.1	3
*A. phagocytophilum*	100.00	China	KF569909.1	2
*E. canis* (Bovine *Anaplasma* sp.)	97.38	Iraq	MN227484.1	1

Abbreviations: PI, percentage identity (similarity in %) between the field isolate from this study and matched species in the GenBank.

**Table 4:** Prevalence of cattle co-infections with pathogen species from the two groups *Theleria*/*Babesia* and *Anaplasma*/*Ehrlichia* by conventional PCR and sequencing in the Rabat-Sale-Kenitra region, Morocco (total number of co-infections equals 84).

Co-Infection	Number of Positives	Prevalence (%)
*T. annulata, A. phagocytophilum*	20	23.8
*T. annulata, A. marginale*	16	19.0
*T. buffeli, A. marginale*	15	17.9
*T. orientalis, A. marginale*	9	10.7
*T. annulata, A. capra*	5	5.9
*T. orientalis, A. capra*	4	4.8
*T. annulata, A. platys*	3	3.6
*T. buffeli, A. capra*	3	3.6
*T. orientalis, A. phagocytophilum*	3	3.6
*T. annulata, A. bovis*	2	2.3
*T. buffeli, A. phagocytophilum*	2	2.3
*T. buffeli, A. bovis*	1	1.2
*T. orientalis, A. platys*	1	1.2

**Table 5:** Risk factor analysis for infection with *A. marginale* and *T. annulata* detected by conventional PCR and sequencing in cattle from the Rabat-Sale-Kenitra region, Morocco.

		*A. marginale*		*T. annulata*	
Variable	Level	Percentage Infection (Lower, Upper)	*p*-Value	Percentage Infection (Lower, Upper)	*p*-Value
Location	ACH	58 (44; 71)	0.0001	0 (0; 100)	<0.0001
	KHM	41 (29; 54)		3 (0; 13)	
	OLM	48 (35; 61)		1 (0; 11)	
	ARB	57 (44; 69)		22 (13; 34)	
	JEM	24 (15; 36)		43 (32; 55)	
	SAY	23 (14; 35)		20 (11; 31)	
	ROM	50 (34; 65)		17 (8; 31)	
	SKH	55 (43; 66)		24 (15; 36)	
	SYZ	48 (35; 60)		41 (29; 54)	
Sex	Female	81 (71; 88)	<0.0001	4 (0; 100)	0.0001
	Male	36 (32; 41)		0 (0; 100)	
Cow type	Beef	80 (72; 87)	0.0001	0 (0; 100)	<0.0001
	Dairy	33 (28; 38)		5 (0; 100)	
Farm type	Modern	75 (67; 82)	0.0001	0 (0; 100)	<0.0001
	Traditional	32 (27; 38)		6 (0; 100)	
Health status	Healthy	44 (39; 48)	0.4121	3 (0; 100)	0.0255
	Sick	54 (31; 76)		12 (0; 100)	
Breed	Cross-breed	47 (42; 53)	0.0295	5 (0; 100)	0.0010
	Local breed	34 (25; 44)		0 (0; 100)	
Age		−0.007 (0.003)	0.0269	0.002 (0.003)	<0.0001
Number of ticks		−0.042 (0.020)	0.0369	0.073 (0.022)	0.0011

Explanation: (Lower, Upper) gives the lower and upper bound of the 95% confidence interval.

**Table 6:** Study locations and the number of collected blood samples per location.

Zone	Study Locations	Number of Blood Samples
Middle Atlas mountains	Ouelmes (OLM)	65
	Ait Ichou (ACH)	48
	Khemisset (KHM)	58
Sub-total		171
The Gharb Plains	Jorf El Melha (JEM)	56
	Sidi Ayach (SAY)	57
	Arabaoua (ARB)	55
Sub-total		168
The Coastal Plains	Skhirat (SKH)	60
	Sidi Yahya Zaer (SYZ)	57
	Rommani (ROM)	52
Sub-total		169
Total		508

**Table 7:** Selected primer pairs and annealing temperature for the detection of the mitochondrial target regions for the genera *Theleria*/*Babesia*, *Anaplasma*/*Ehrlichia*, and *Rickettsia* primers in cattle from Rabat-Sale-Kenitra region, Morocco.

General Target	Forward Primer 5′→3′	Reverse Primer 5′→3′	Fragment Size (bp)	Annealing T (°C)	Reference
*Theileria*/*Babesia* spp. (18S rRNA) (RLB-F2/R2)	GACACAGGGAGGTAGTGACAAG	CTAAGAATTTCACCTCTGACAGT	460–500	58	[53]
*Anaplasma*/*Ehrlichia* spp. (16S rRNA) (Ehr-16S-D1/R)	GGTACCTAYAGAAGAAGTCC	TAGCACTCATCGTTTACAGC	345	54	[76]
*Rickettsia* spp. (16S rRNA) (Rick-F1/R2)	GAACGCTATCGGTATGCTTAACACA	CATCACTCACTCGGTATTGCTGGA	350–400	60	[53]

Abbreviations: T, temperature.

**Table 8:** GenBank accession numbers assigned to acquired field isolates from Rabat-Sale-Kenitra region, Morocco.

Pathogen	Accession Number
*Theileria annulata*	OL305716
*Theileria buffeli*	OL305720 OL305719 OL305723
*Theileria orientalis*	OL305721 OL305718
*Babesia bovis*	OL305722 OL305724
*Babesia occultans*	OL305717
*Anaplasma marginale*	OK606081 OK606082
*Anaplasma phagocytophilum*	OK606078 OK606088 OK606076 OK606072 OK606077 OK606073
*Anaplasma capra*	OK606083 OK606084 OK606089
*Anaplasma platys*	OK606071
*Anaplasma bovis*	OK606086 OK606085
*Bovine Anaplasma* sp.	OK606070
*Ehrlichia minasensis*	OK606068

## Data Availability

The DNA sequences generated during this study are openly available in GenBank. Other primary data are available on request from the corresponding author.
